# Reproductive biology and genetic diversity of the green turtle (*Chelonia mydas*) in Vamizi island, Mozambique

**DOI:** 10.1186/2193-1801-3-540

**Published:** 2014-09-19

**Authors:** Rita Anastácio, Camila Santos, Cardoso Lopes, Helena Moreira, Luis Souto, Jorge Ferrão, Julie Garnier, Mário J Pereira

**Affiliations:** Departamento de Biologia e CESAM, Universidade de Aveiro, 3810-193 Aveiro, Portugal; Maluane - Cabo Delgado Conservation and Tourism, Pemba, Mozambique; Lúrio University, Nampula, Mozambique; The Zological Society of London, Regent’s Park, London, NW1 4RY UK; AFPR - A For Plankton Research, 3800-365 Aveiro, Portugal

**Keywords:** *Chelonia mydas*, Reproductive behavior, mtDNA diversity, Climate changes, Mozambique Channel (MZC)

## Abstract

**Introduction:**

Vamizi, an Island located in the Western Indian Ocean, is visited by a small and not fully characterized green turtle (*Chelonia mydas* (L.)) population. This population is threatened by natural hazards and several human activities, which are used to identify conservation priorities for marine turtles.

It was our aim to contribute to the knowledge of marine turtles that nest in Vamizi, with respect to its regional management, and to an area that may possibly be included on the UNESCO World Heritage List due to its potential Outstanding Universal Value.

**Case description:**

Here, we evaluate the nesting parameters (incubation period, clutch size, hatching and emergence successes rates) and patterns over an 8-year (2003 – 2010) conservation program. We also present the results of genetic diversity based on the analysis of approximately an 850 pb fragment of the mitochondrial DNA control region.

**Discussion and evaluation:**

We found that Vamizi beaches host a small number of nesting females, approximately 52 per year, but these have shown a reduction in their length. High hatching success (88.5 ± SD 17.2%, *N* = 649), emergence success rates (84.5 ± SD 20.4%, *N* = 649) were observed, and genetic diversity (*N* = 135), with 11 haplotypes found (7 new). It was also observed, in the later years of this study, a reduction in the incubation period, a dislocation of the nesting peak activity and an increase in the number of flooded nests and an increase of the number of nests in areas with lower human activity.

**Conclusions:**

Some resilience and behavioral plasticity seems to occur regarding human territory occupancy and climate changes. However, regardless of the results, aspects like what seems to be the reduction of some cohorts, the number of flooded nests and the diminishing of the incubation period (East and South facing beaches), show that conservation efforts have to be improved.

**Electronic supplementary material:**

The online version of this article (doi:10.1186/2193-1801-3-540) contains supplementary material, which is available to authorized users.

## Introduction

Mozambique (MZ) possesses a vast coastline and several islands that are used as nesting sites for a number of species of turtles. Five marine turtles species are known to nest along the coast of MZ, green (*Chelonia mydas* (L.)), hawksbill (*Eretmochelys imbricata* (L.)), loggerhead (*Caretta caretta* (L.)), olive ridley (*Lepidochelys olivacea* (Eschscholtz)) and leatherback (*Dermochelys coriacea* (L.)) (Hughes 1971; Costa et al. 2007; Videira et al. 2008).

The green turtle is widespread (Hughes 1971; Videira et al. 2008) and occupies several marine habitats dispersed over extensive areas (Tröeng et al. 2005; Piniak and Eckert 2011; Blanco et al. 2012). In MZ the species nests in cape São Sebastião (Narane 2008a), Bazaruto Archipelago (Narane 2008b) (22°10’S) and at the north region (Hughes 1971; Costa et al. 2007), with the majority found in the Quirimbas Archipelago (Videira et al. 2008). Vamizi is one of the largest islands of the MZ Quirimbas Archipelago and it has been an observed rookery for green and hawksbill turtles (see Garnier et al. 2012).

Though reproductive females migrate hundreds to thousands of kilometers between rookeries and feeding grounds (Limpus 2008; Godley et al. 2010; Tröeng et al. 2005), they are known to show some fidelity to their nesting grounds (Meylan et al. 1990; Lee et al. 2007; Limpus 2008). Some of the most important green turtle rookeries in the Western Indian Ocean (WIO) have been previously described. At the Eparses Islands of Europa, Tromelin and Grande Glorieuses, green turtle populations have been monitored since the 1980’s (Lauret-Stepler et al. 2007). Other studies include those at Juan de Nova (Lauret-Stepler et al. 2010) and Mayotte Island (Comoros Arquipelago) (Bourjea et al. 2007a). However, information on nesting turtles is either sparse or lacking in other adjacent countries (Mortimer 2002), especially in Mozambique, Madagascar and Somalia, where this species is vulnerable to human activity (Shanker 2004; Mortimer 2002; Bourjea et al. 2008). Studies of tracked nesting green turtle females have revealed the migratory pathways of these females in the WIO, showing that they use the Madagascar coast as foraging ground, as well as Mozambique, Kenya, Tanzania and Somalia coasts (Bourjea et al. 2013). The study identifies two oceanic corridors (one in the north of the Mozambique Channel (11°S - 14°S) and the other at the south of the Mozambique Chanel (17°S - 23°S) from the north of Europa to the north of MZ (38°E - 41°E)) as well as two coastal corridors (one at the east African coast, between Mozambique and Tanzania (16°S - 7°S), and the other across the west coast of Madagascar), which emphasizes that the extreme north of Madagascar is an important coastal migratory corridor (Bourjea et al. 2013). Using satellite transmitters, Garnier et al. (2012) showed the migration routes of four green turtle females tagged in Vamizi travelling to foraging grounds in Tanzania, Kenya and northwest Madagascar (Nosy Makamby).

To further our knowledge on the movement of turtles, investigators have been using tracking approaches but also molecular analysis (Lee 2008). Molecular methods have been used with the aim to better understand the life cycle of green turtles. With the use of molecular markers, such as mitochondrial DNA (mtDNA), it has been possible to understand aspects of their biology such as: natal origins and connection to foraging grounds (Meylan et al. 1990; Lahanas et al. 1998; Dutton et al. 2008), population structure (Encalada et al. 1996; Bjorndal et al. 2005; Formia et al. 2006, 2007), and phylogeography (Avise et al. 1992; Encalada et al. 1996; Formia et al. 2006; Bourjea et al. 2007b). Comprehension of the genetic diversity and structure of each population is important, especially for biodiversity managers who use that information to define conservation units to protect (Bagda et al. 2012). For the WIO, the latest published data about mtDNA haplotypes are from Formia et al. (2006) and Bourjea et al. (2007b). For the Vamizi rookery, however, there is currently no molecular information published in the literature. We suggest that owing to its context, molecular data from the Vamizi rookery may contribute important insights for green turtle conservation and management.

The aim of this study was to provide information on green turtle nesting activity and seasonality at the Vamizi rookery. We also analysed mtDNA control region sequences of *C. mydas* to provide information on its genetic diversity. An additional aim was to explore possible changes in nesting due to climate and anthropogenic pressures.

## Methods

### Study area

**Figure 1:**
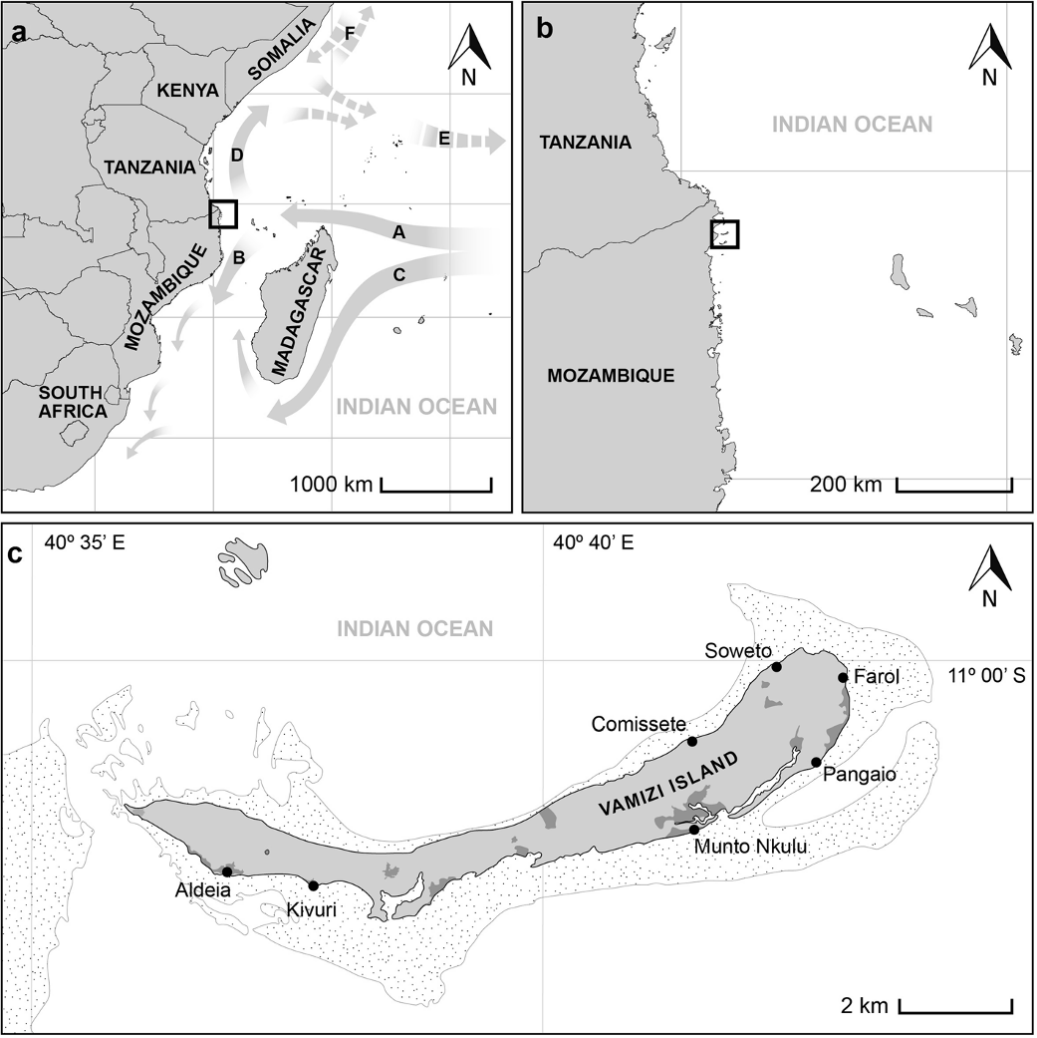
**Vamizi Island, its beaches, and its location in the Quirimbas Archipelago, Cabo Delgado Province on the northern Mozambique coast. a** Currents: A – South Equatorial Current (SEC); B – Mozambique Current; C – Madagascar Current; D – East African Coastal Current; E – Equatorial Counter Current (Nov–April); F – Somalia Currents. **b** Vamizi location in the MZ Channel. **c** Vamizi island with main beaches (a adapted from Richmond 2002). **b, c** adapted from Missão Hidrográfica de Moçambique MHM Missão Hidrográfica de Moçambique MHM 1974). Redrawn with Adobe Illustrator C.S.5.5 program. North facing beaches: Comissete (11° 00′ 54″ S, 40° 41′ 23″ E), Soweto (11° 00′ 08″ S, 40° 42′ 17″ E); East-South facing beach: Farol (11° 00′ 17″ S, 40° 42′ 55″ E); South facing beaches; Pangaio (11° 01′ 07″ S, 40° 42′ 39″ E); Munto Nkulu (11° 01′ 44″ S, 40° 41′ 33″ E); Kivuri (11° 02′ 18″ S, 40° 37′ 44″ E); Aldeia (11° 02′ 11″ S, 40° 36′ 57″ E).

The study area was Vamizi Island (Figure 1), a 12 km long, 0.5–2.0 km wide land mass situated at the north of the Quirimbas Archipelago (a chain of 32 islands) at the northwest edge of the Mozambique Channel (MZC). It belongs to the WIO Ecoregion 95 designated as the “East Africa Coral Coast” by the Marine Ecoregions of the World (MEOW) classification scheme (Obura et al. 2012). The island belongs to the specific location named “Northern Mozambique to southern Tanzania – Nacala – Quirimbas – Mtwara” (Obura et al. 2012) with potential Outstanding Universal Value (OUV) to be considered as a World Heritage site.

**Figure 2:**
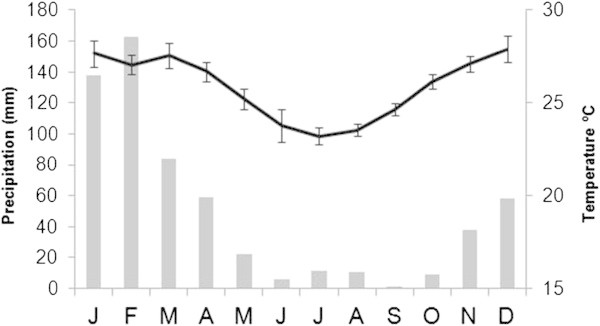
**Climatogram of Pemba with averages between 2004 and 2010, obtained from National Oceanic and Atmospheric Administration (NOAA,** http://www.noaa.gov/index.html **).**

Vamizi has monsoon seasonality with variation of temperature and rainfall, which is likely enhanced by mesoscale dynamic eddies. McClanahan’s (1988) review of seasonality patterns in east Africa’s coastal waters, focusing on the area 10° north and south of the equator, stated that the division between northeast (NE) (October/November to March) and southeast (SE) monsoons (March to October) are indicative of two coastal seasons, which affect oceanographic processes (physical, chemical, biological). The SE monsoon has lower air temperatures which lower surface seawater temperatures. Also, wind run and speed are greater during SE monsoons and, as a consequence, current speed and water column mixing are higher (McClanahan 1988). The monsoon seasonality of the Indian Ocean, considered as a strong ocean-climate interaction, does strongly modulate current speed and variability of the south MZC (Obura et al. 2012). Yet, at the north of the Channel, the influence of the monsoon northeast winds dominates along the northern coast of Mozambique (e.g. Pemba, 13°S) in the austral summer (NE monsoon), affecting Vamizi, and southwest winds dominate during the austral winter (SE monsoon) (Ternon et al. 2014; see Climatogram of Pemba for the period 2004 – 2010 at Figure 2).

Vamizi has small air temperature amplitudes and considerable variation in precipitation values. The period 2004 – 2010 had lower rainfall averages of below 50 mm, with 2005 being particularly dry with an average rainfall of 22.6 mm, and 2006, 2008 and 2010 having higher average precipitation of 85.4, 68.4 and 73.7 mm, respectively.

These waters receive the South Equatorial Current (SEC). After reaching the African coast, the NMC splits into the East African Coastal Current (EACC) and a southerly branch (Mozambique Current) that flows into the northern MZC (Figure 1) (for details, see Ternon et al. 2014). The water circulation in the MZC is highly variable and eddy driven. These eddies have a strong impact on food webs, especially affecting top-level consumers such as turtles, seabirds and marine mammals (Obura et al. 2012).

### Monitoring program and data collection

#### Field effort

The monitoring program in Vamizi Island started in September 2002 with daily foot patrols conducted by monitors. Each team comprised at least, three trained scouts and the information was gathered daily following the standard protocols (Eckert et al. 1999).

Comissette and Farol beaches have been monitored in day patrols since October 2003 and night patrols since January 2004, every hour for at least 3 months (Table 1) during the peak of the nesting activity. This night monitoring was conducted by teams of 3–4 members that were responsible for tagging and for gathering information on emergences, nest attempts, species identification and specimen size.

**Table 1:** **Field effort, months with night-time patrols**

2004	2005	2006	2007	2008	2009	2010
March – July	March – July	February – July	January – December	January – August	February – September	February – July

Every morning at the day patrols, activity was checked above the high tide line. The team recorded tracks, species identification (based on tracks type) and nesting activity (differentiating between nesting and non-nesting emergences). For any new nests, monitors recorded their GPS coordinates and then marked them with bamboo poles behind the nest. In addition to recording nesting date, monitors gathered associated information of hatching activity, such as hatching date, excavation date, and other nesting parameters (hatched at the nest and/or undeveloped (not before 90 days after eggs laid)) to evaluate hatching success.

Following Schroeder and Murphy (1999) a “crawl” was interpreted as “tracks and other sign left on a beach by a sea turtle”; a “false crawl” was interpreted as “a crawl resulting from an abandoned nesting attempt (a non-nesting emergence)”.

#### Biometric information

Identification (Pritchard and Mortimer 1999) and the biometric information, made in triplicate after egg deposition, is represented by CCL (minimum curved carapace length) and CCW (curved carapace width) lengths from the observed turtles (following Bolten (1999) methodology) and by size and shape of the tracks (Pritchard and Mortimer 1999; Schroeder and Murphy 1999).

#### Tagging

Turtles were tagged, according to the methodology described by Balazs (1999). The examination for tags occurred during night patrols. Titanium tags (http://www.stockbrands.com.au/titanium.html) (Stockbrands Pty Ltd., Perth, Australia, http://www.stockbrands.com.au) were applied at proximal end of both front flippers. The presence of tags (tag series MZC 0000 – MZC 0999) was recorded. Missing tags were replaced or applied if not previously tagged. The first external tags were applied on 18^th^ March 2004 (MZC 0004/MZC 0005; turtle ID VZ001). For all turtles captured, date, site, tag number and activity were recorded. The individuals’ location (latitude and longitude) was recorded with a GPS (Magellan NAV5000D, used in 2D non-differential mode).

#### Tissue collection

Samples were taken following recommendations of Dutton (1996) between 2008 and 2010 by biopsy punch (approximately 5 mm^3^) from the extremity of back flippers from adult females (*N* = 63) or dead hatchlings. All samples were registered with date of collection, tag number and beach ID. Samples (*N* = 135) were from six nesting beaches and were stored in ethanol 70%, and frozen in 1.5 mL eppendorf tubes.

### Mitochondrial DNA control region extraction, amplification and sequencing

Samples were obtained to determine the genetic diversity of the green turtles that use Vamizi Island as a rookery, and to compare this with other populations/subpopulations. DNA extraction was performed following the standard phenol/chloroform procedure (Sambrook et al. 1989) with some modifications and Chelex procedure (Walsh et al. 1991). A 1000 bp-fragment of the mitochondrial DNA control region was amplified via PCR in a Bio-Rad iCycler Thermal Cycler (Hercules, CA, USA), using LCM15382 (5′-GCT TAA CCC TAA AGC ATT GG-3′) and H950g (5′- GTC TCG GAT TTA GGG GTT TG-3′) (Lara-Ruiz et al. 2006, Abreu-Grobois, F.A. *pers. comm.*, abreu@ola.icmyl.unam.mx) primers. PCR conditions (for 25 μL: 2.5 μL buffer containing 1.5 mM of MgCl_2_, 0.5 μL dNTP (200 μM), 1 μL (0.4 μM) each primer, 0.5 μL (2.5 U) Taq DNA Polymerase, 1 μL (2 ng) DNA and 18.5 μL H_2_O) for these primers were as follows: initial denaturation of 5 min at 94°C, followed by 36 cycles of 30 s at 94°C, 30 s at 50°C and 1 min at 72°C, and a final extension step of 10 min at 72°C. Amplification was verified by electrophoresis of 6 μL of each reaction product in 2% agarose gel and a Transiluminator UVP Bio Doc-It™ System. Amplicons were sequenced in a company: PCR product was sequenced using the BigDye® Terminator v3.1, Cycle Sequencing Kit (Applied Biosystems; Princeton, USA). Purification was done through gel filtration, using Centri-Sep™ 96-Well Plates (Applied Biosystems; Princeton, USA). Sequence detection was done on an automatic sequencer ABI PRISMR 3730XL Genetic Analyser (Applied Biosystems; Princeton, USA).

### Data analysis

For determining of nesting parameters we used samples from Comissete, Farol, Pangaio, Munto Nkulu and Soweto beaches (Figure 1). For genetic diversity analysis we used samples of tissue from Comissete, Farol, Pangaio, Kivuri, Aldeia and Munto Nkulu beaches.

#### Reproductive biology – nesting parameters

Nesting parameter averages were obtained using records of all beaches combined and per beach. The parameters of Farol and Comissete were given emphasis because these beaches represent 54.7 and 28.8%, respectively, of the total records of our sample.

Using the entire database (*N* = 1303), we counted the amount of records per year in percentages for the two main beaches, to obtain polynomial tendency lines.

##### Nesting success

The nesting success was defined as “the proportion of nesting activities that resulted in a nest” (Godley et al. 2001).

##### Inter-nesting period and remigration interval

The inter-nesting period and remigration interval were obtained using the records of tagged females that visited Vamizi in the sampled beaches between November 2004 and October 2010. Following Bourjea et al. (2007a) the mean inter-nesting interval was calculated as the mean of all observed inter-nesting intervals from the records of tagged turtles, after excluding intervals <7 days. These were considered to be unsuccessful nesting events. The remigration interval was obtained from records of tagged females that visited Vamizi in different nesting seasons and years and was defined as “the period, in years, between nesting seasons for an individual female.” (Alvarado and Murphy 1999).

##### Nesting females and clutch frequency

We estimated the number of nesting females per year based on the number of tagged turtles and on the observed clutch frequency (obtained as the average number of nests laid per tagged female per year) and on the total number of nests laid per year (Alvarado and Murphy 1999).

##### Clutch size, hatching and emergence successes

Clutch size, hatching and emergence successes were determined following the methodology described by Miller (1999) using records from all beaches. We calculated the number of eggs laid per year, average of nests/year and average of nests/month. Hatching success was defined as “the proportion of hatchlings that hatched out of their shells respectively” (Miller 1999). Emergence success was defined as “the proportion of hatchlings that reached the beach surface” (Miller 1999). The formulas used to calculate the clutch size, hatching and emergence successes were described by Miller (1999).

##### Incubation period

The incubation period was obtained using all records from all the sampled beaches and according to the date when the nest was laid.

#### Flooded nests

The percentage of flooded nests per year was generated from the records considering that a nest was considered to be flooded when it had been completely over washed.

#### Statistical analysis

Our initial sample included 1303 records, registered between 2002 and 2010, corresponding to observations *in situ* of adult green turtles (nesting and doing other activities) in all beaches combined. Data from 2002 were scarce, corresponding to a different field effort from the 2003 – 2010 period.

All statistical analyses were performed on PASW Statistics 18 and the Microsoft Office Excel 2007 Programs. Significance was estimated at the 95% confidence level. Variables like incubation period, clutch size, hatching and emergence successes were compared between years (data between January and July) using One-Way ANOVA and the post-hoc tests of Games Howell, Tukey or Scheffe, when statistically significant differences were detected (p < 0.05).

#### Genetics diversity and phylogenetic analysis

Sequence alignments were performed with the software CLUSTAL W software version 1.3.1.1 (Thompson et al. 1994) and nucleotide analysis with BioEdit Alignment Editor v.7.0.9 (http://www.mbio.ncsu.edu/BioEdit/bioedit.html). Basic Local Alignment Search Tool (BLAST) search was used to verify existing similarities with deposited sequences in the GenBank database (http://blast.ncbi.nlm.nih.gov/Blast.cgi). New sequences were deposited (3, 9.05.2011) in the GenBank database under the accession number JF926556, JF926557, JF926558, JF926559, JF926560, JF926561, JF926562.

The network tree of the haplotypes was built by the median-joining (MJ) method (Hamabata et al. 2009) using Phylogenetic Network Constructions version 4.6.0.0 (http://www.fluxus-engineering.com).

Haplotype (*h*) diversity was obtained using DnaSP v. 5 (Monzón-Argüello et al. 2010).

## Results

### Turtle biometry

**Figure 3:**
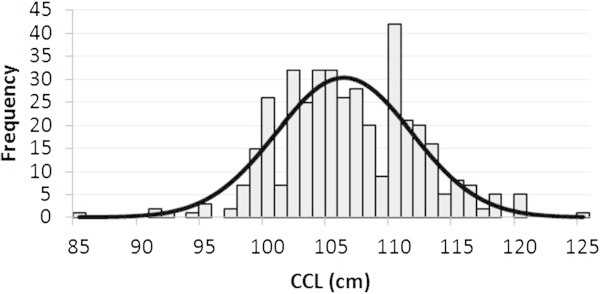
**Distribution of average minimum curved length, CCL (** ***N*** **=401).**

The CCL average of the turtles measured on night patrols (2004 – 2010) was 106 ± 5 cm (*N* = 401) ranging from 85 to 125 cm (Figure 3), and the CCW average was 99 ± SD 6 cm (*N* = 398) ranging from 76 to 120 cm.

This distribution (Figure 3) shows the occurrence of specimens of several sizes/ages but also indicates that several cohorts where removed with particular incidence in thus below 110 cm. The CCL shows, in yearly average, a reduction from 108 ± 5 cm (*N* = 38) in 2004, to 105 ± 4 cm (*N* = 49) in 2010.

### Reproductive biology – nesting parameters

The average number of green turtles that visited Vamizi was 162.9 ± 44.9 individuals.year^-1^ (*N* = 1303). The average annual number of nests was 130.00 ± 32.7 (*N* = 1040) ranging from 79 nests (2004) to 173 (2008).

#### Nesting success

**Figure 4:**
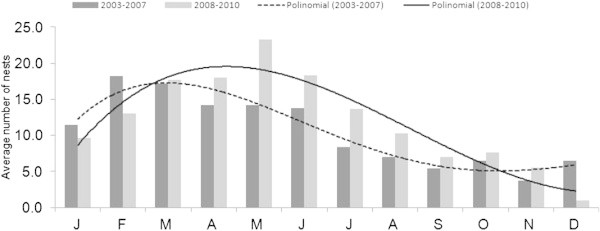
**Nesting activity (mean ± SD) by month in sampled beaches combined, in the period 2003 – 2010 with polinomial tendency lines for two periods of time: 2003 – 2007 and 2008 – 2010.**

The nesting success was above 73% for all years. The highest average of nests.month^-1^ (all sampled beaches combined) peaked in May with 17.6 ± SD 9.9 nests (*N* = 141) in 2003 – 2010. The first semester shows the highest nesting activity (Figure 4).

**Figure 5:**
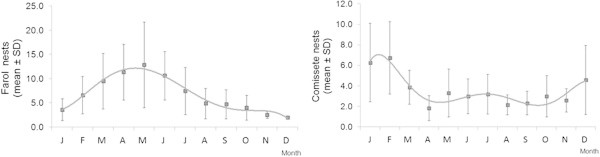
**Mean green turtle nests numbers by month in Farol and Comissete beaches for the period from 2004 to 2010 (with polynomial tendency lines).**

**Figure 6:**
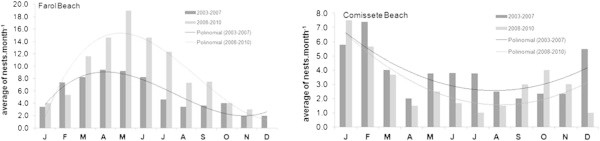
**Nesting activity in Farol beach (left) and Comissete beach (right), in the period 2003 – 2010 with polinomial tendency lines for two periods of time: 2003 – 2007 and 2008 – 2010.**

**Figure 7:**
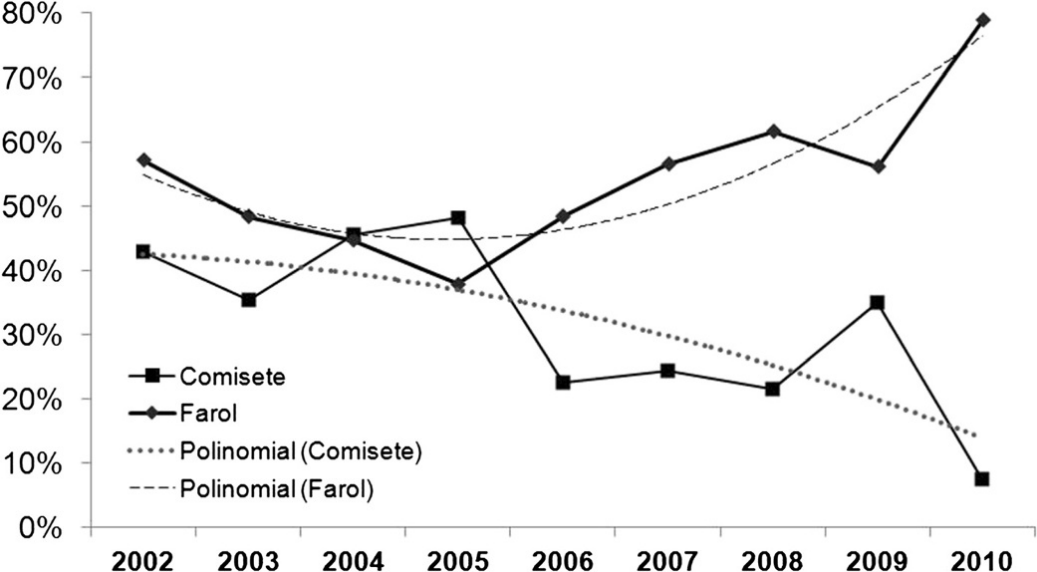
**Percentage of records by year between 2002 and 2010.** The number of records is proportional to the number of nests and emergences.

**Figure 8:**
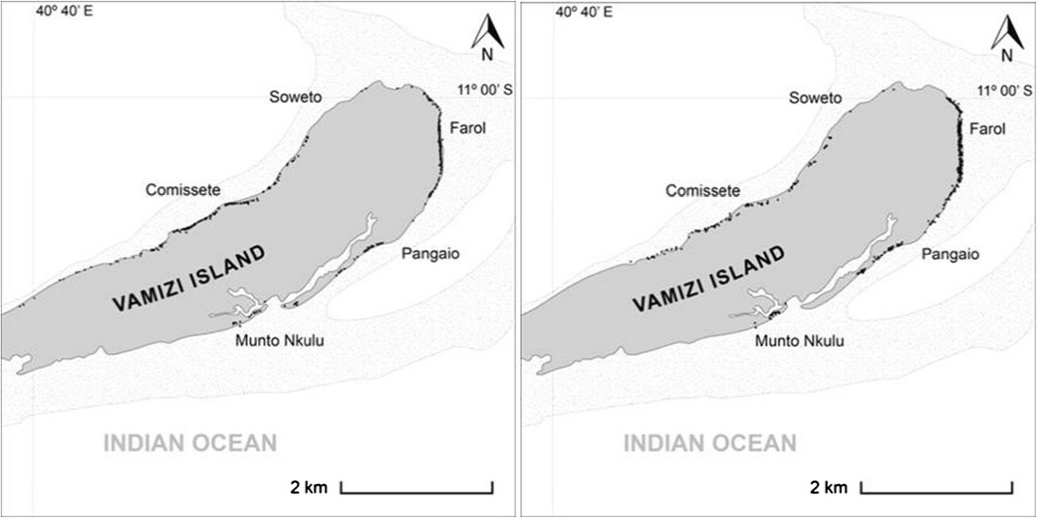
**Distribution of nests on Comissete and Farol showing before 2005 (left) and after 2005 (right).**

Because Farol and Comissete beaches combined represent 83.5% of the sample, the nesting activity of both beaches was analyzed.The average number of nests was, higher at Comissete beach between October/November and February/March, which is different from what was recorded at Farol beach (Figure 5), where the nesting activity occurs all year with higher activity between February and July.It’s also possible to verify, for Farol (Figure 6), a dislocation of the nesting peak from April (2003 – 2007) to May (2008 – 2010).When examining nest records over time in Farol and Comissete beaches, a polynomial tendency (Figure 7) demonstrates that since 2005, records diminished at Comissete and increased in Farol.The distribution and reduction of the number of records at Comissete is also shown in Figure 8. After 2005, the majority of nests were concentrated in Farol.

#### Inter-nesting period and remigration interval

Between March 2004 and August 2010, 161 *Chelonia mydas* turtles were tagged on night patrols.

**Figure 9:**
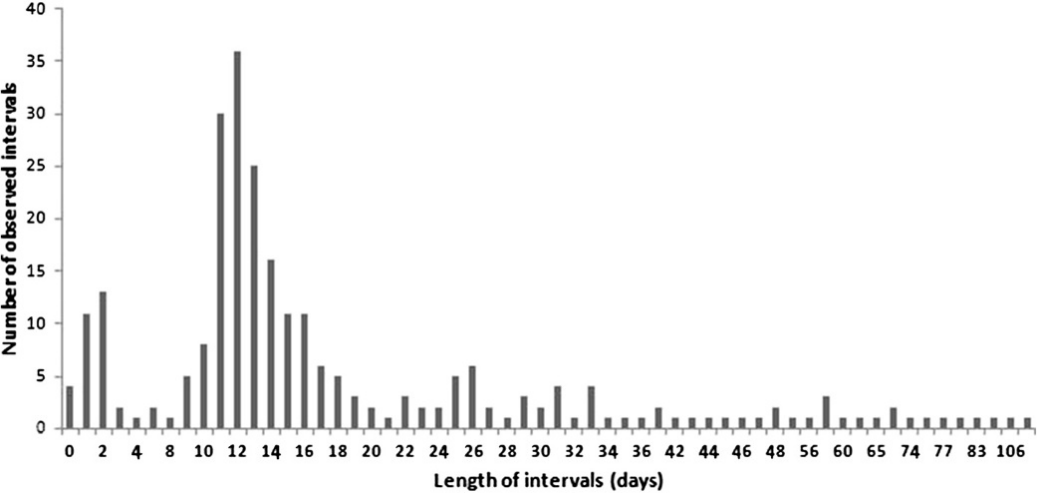
**Intervals separating nesting attempts.**

The modal observed inter-nesting interval was 12 days (Figure 9), with a mean interval of 20.3 ± SD 15.8 days (range 8 – 90 days, *N* = 259). Approximately 88% of the tagged turtles made an emergence at Vamizi in less than 34 days after their first emergence. Approximately 10.8% came to the beach 0 – 2 days after their first emergence. The majority (59.1%) re-emerged between 9 and 18 days after. From our tagged sample, the same turtle emerged at Vamizi between 2 and 7 times (in maximum) per year.

Table 2 shows the numbers of emerged/tagged/returned turtles on all the beaches sampled. Of these, 32.9% re-emerged at Vamizi in following years, 84.0% of which did so it on the same beach, thus showing philopatry.

**Table 2:** **Number (#) of emerged, tagged and returned turtles per year (night patrols)**

Year	# Tagged females. Year ^-1^	Returned females marked that returned in subsequent years
2004	13	4
2005	19	9
2006	21	6
2007	23	6
2008	41	–
2009	22	–
2010	22	–

The remigration interval was 2.6 ± SD 1.1 years (range 222 – 1518 days; *N* = 30).

#### Nesting females and clutch frequency

Table 3 shows the observed clutch frequency for the latest three years of the program. The year 2009 shows the lower estimated value of 33.4 nesting females.year^-1^.

**Table 3:** **Months of night-time monitoring, number (#) of adult female green turtle tagged, number of clutches laid by tagged females, observed clutch frequency ± SD, on beaches combined for Vamizi Island**

	2008	2009	2010	All
Months	January-August	February-September	February-July	
**# of nesting females tagged**	41	22	22	85
**# of clutches laid by tagged females (%)**	3.25	3.44	2.65	
	65 (36%)	62 (54%)	53 (30%)	180
**Observed clutch frequency (CF)**	3.3	3.4	2.7	3.4
**Estimated # of nesting females.year** ^**-1**^	55.7	33.4	66.4	

#### Clutch size, hatching and emergence successes

The average clutch size (all beaches combined, 2003 – 2010) was 116.7 ± 26.5 eggs (*N* = 649). Comissete beach had an average clutch size of 120.2 ± 31.5 eggs (*N* = 177), and Farol beach has an average clutch size of 114.2 ± 24.1 eggs (*N* =371).

The overall mean hatching success was 88.5 ± SD 17.2% (*N* = 649) and the overall mean emergence success was 84.5 ± 20.4% (*N* = 649). The year 2003 showed a hatching success of 75.3 ± 33.0%, in contrast with other years, which showed higher hatching success rates (>80%).

One–Way ANOVAs show significant differences in the overall mean hatching success between 2005 and 2009 (F_7,595_ = 3.077 p < 0.003), and also show significant differences for the overall mean rates of emergence success (F_7,595_ = 5.017 p < 0.001), between 2004/2005 (Games-Howell post hoc test p = 0.033), 2005/2009 (p < 0.001), and 2005/2010 (p = 0.013).

For Farol beach there were no differences in hatching success (F_7,353_ = 1.503, p = 0.165). For emergence success (F_7,353_ = 3.489, p = 0.001) the years of 2004/2005 (post-hoc test Games-Howell: p = 0.025), 2005/2009 (post-hoc test: p = 0.004), and 2005/2010 (post-hoc test: p = 0.020), were significantly different.

There were no differences in hatching success (F_7,142_ = 0.884, p = 0.521), and emergence success (F_7,142_ = 0.788, p = 0.598) between years for Comissete beach.

#### Incubation period

The mean incubation period (i.p.), across all observed beaches was 64.4 ± 12.3 days (*N* = 687; 2003 – 2010). The overall annual i.p. average reveals a pattern: a smaller value in a year is always followed by a peaking value in the next year. However, the difference between the last 2 years isn’t significant (60.7 ± 7.4 to 61.7 ± 9.3 days).

Significant differences in the overall i.p. mean are almost always consistent, between two consecutive years (2004 and 2005; 2005 and 2006; 2008 and 2009). One–Way ANOVA (F_7,631_ = 6.619 p < 0.001) shows no significant differences in the overall i.p. mean between the years of 2004, 2006 and 2008, but they are significantly different from the other years of the study. For example, 2004 is significantly different from 2005 and 2009 (Games-Howell post hoc test p = 0.049; p = 0.014); 2006 is significantly different from 2005 (p = 0.001), 2009 (p < 0.001) and 2010 (p = 0.002), which were hot years.

The overall averages of i.p. in Vamizi beaches are as follows: North facing beaches – Comissete 62.1 ± 11.4 days (*N* = 182) and Soweto beach, 57.8 ± SD 23.6 days (*N* = 6); East-South facing beach – Farol beach, 65.5 ± 11.8 days (*N* = 392); South facing beaches – Munto Nkulo beach, 65.1 ± 13.3 days (*N* = 42); Pangaio beach, 64.7 ± 15.0 days (*N* = 65).

**Figure 10:**
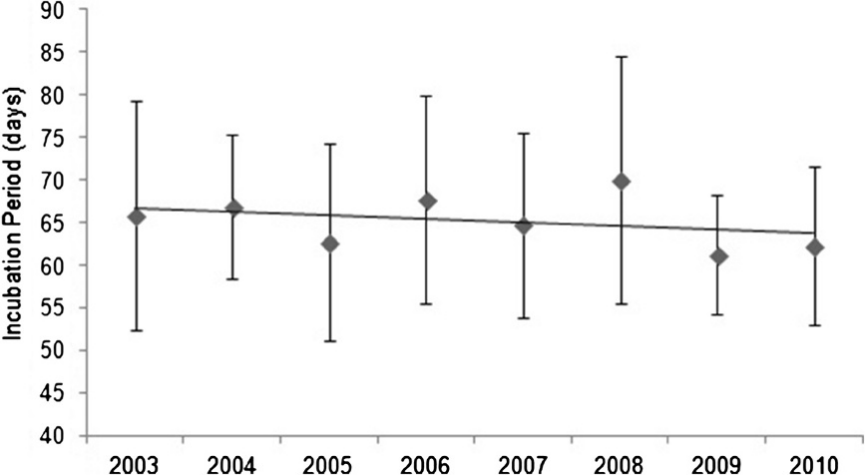
**Patterns of incubation period in Farol beach from 2003 to 2010 (** ***N*** **= 392).**

At Farol beach the i.p. (F_7,372_ = 4.660, p < 0.001) was significantly different between 2005/2006 (post-hoc test Games-Howell: p = 0.049), 2005/2008 (post-hoc test: p = 0.001), 2008/2009 (post-hoc test: p = 0.001), and 2008/2010 (post-hoc test: p = 0.003). There is a small decreasing tendency of the ip of Farol beach over time (Figure 10) indicating possible increases in air/sand temperature. There were no differences in i.p. (F_7,147_ = 2.960, p = 0.006) between years for Comissete beach.

### Flooded nests

Nest losses occur because of flooding and predation. Predation, however, appears to be very low at Vamizi, since an average of 0.74 ± 2.35 eggs (*N* = 649) were lost to predation. Conversely nests lost because of flooding were greatest in 2007 (51.2%), 2009 (29.9%) and 2010 (38.0%). Also, February, March, August and September are the months when percentages of flooded nests rise above 25%.

### Genetics diversity and phylogenetic analysis

Within the analyzed region, 74 polymorphic sites were found (Additional file 1: Table S1) and 11 mtDNA haplotypes: IND1, IND3, JF926556, JF926557, JF926558, JF926559, JF926560, JF926561, JF926562, CMJ1, CM8, were identified from the 135 samples (Table 4). Four haplotypes described here have been found elsewhere: IND1 and IND3 (GenBank accession n°s. AF529028.1 and AF529030.1, respectively) CM8 (GenBank accession n°. Z50130) and CMJ1 (GenBank accession n°. AB472300.1). JF926556, JF926557, JF926558, JF926559, JF926560, JF926561 and JF926562 are described for the first time for this region (GenBank accession n°. 1452591).

**Table 4:** **Distribution of observed green turtle haplotypes by beach on Vamizi island**

	Total	IND 1	CM 8	IND 3	CMJ 1	JF926558	JF926556	JF926557	JF926559	JF926560	JF926561	JF926562
**Farol**	81	55	8	7	4	2	1	1	1		1	1
**Comissete**	37	25	11							1		
**Pangaio**	9	9										
**Kivuri**	6	3	2			1						
**Aldeia**	1	1										
**Munto Nkulu**	1			1								
**Total (** ***N*** **)**	135	93	21	8	4	3	1	1	1	1	1	1

If IND1 is used as a reference sequence, JF926558, JF926556 and IND3 haplotypes differ by one substitution at base positions 306, 534 and 553 respectively. Haplotypes JF926562 and JF926557 showed three and six substitutions respectively. The remaining five haplotypes are more diverse showing JF926561 one deletion and 10 substitutions, CM8 two deletions and 34 substitutions, JF926560 three deletions and 34 substitutions, JF926559 three deletions and 39 substitutions and CMJ1 two deletions, one insertion and 28 substitutions (Additional file 1: Table S1). Haplotypes CM8 and JF926560 are separated from JF926559 by the same five substitutions, in the base positions 261, 266, 267, 270, 272; only one deletion was detected between CM8 and JF926560.

Vamizi island has high values of haplotype (*h*) diversity, with the most sampled beach (Farol) showing an *h* value of 0.605.

Among the 135 specimens of green turtles IND1 was the most dominant haplotype (Table 4). IND1 occurs in 68.88% of turtles, IND3 in 5.93%, CM8 in 15.56%, CMJ1 in 2.96% and JF926558 in 2.22%. The other haplotypes occurred in 0.74% each (IND1 > > CM8 > > IND3 > > CMJ1 > > JF926558 > > others).

**Figure 11:**
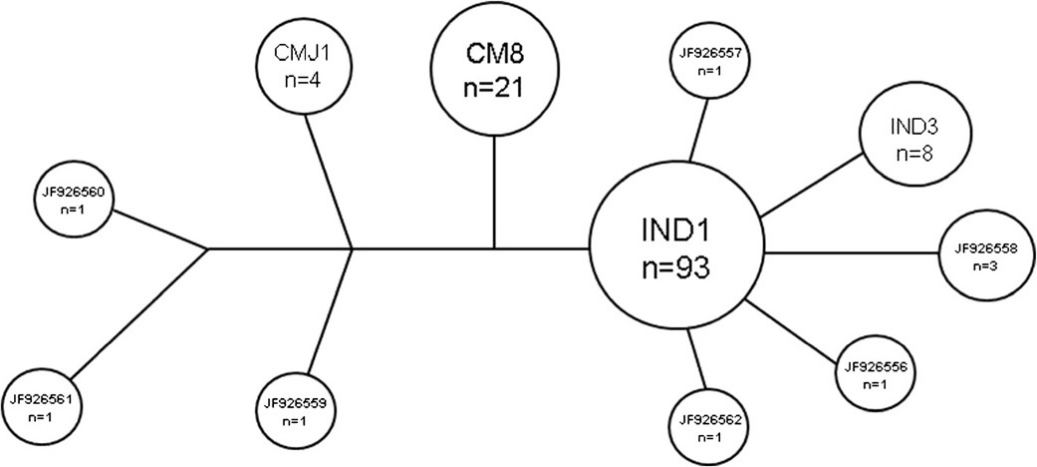
**Network tree based on the 11 identified haplotypes.**

Farol is the most diverse beach (Table 4), exhibiting 10 haplotypes among 81 samples, followed by Comissete (*N* = 37), Kivuri (*N* = 6) with 3 haplotypes each. The remaining places presented only one haplotype.The network tree of green turtles based on the mtDNA control region sequences (Figure 11) shows the relationships among the identified haplotypes.

## Discussion

### Turtle biometry

Based on average CCL, the specimens that nest in Vamizi Island were smaller compared with others that nest in the region. For example, at Mohéli the CCL average is 108.1 ± 5.3 cm (range: 92 – 129 cm; *N* = 742; Innocenzi et al. 2010), and at Juani Island (Tanzania) the CCL average is 107.2 ± 4.7 cm (range: 101–118 cm; *N* = 18; West et al. 2013).

Analysis year by year showed a reduction in size of measured specimens and consequently age, as well as a reduction in the expected abundance of each cohort. Among the various threats to marine turtles, fishing techniques are known to contribute greatly to mortality (Lewison et al. 2004; Wallace et al. 2010). Bycatch has a high impact on marine turtles in the WIO and poaching has been reported to occur in Kenya, Madagascar, Mozambique, Seychelles and Tanzania. All these countries have rookeries or are known as feeding grounds of groups of WIO marine turtles (Bourjea et al. 2008). The diminishing of some cohorts in the Vamizi rookery may indicate the presence of anthropogenic pressures on turtles while at the sea, during the inter-nesting period or between nesting and feeding sites.

### Reproductive biology

There is variability in several nesting patterns over the years that can be discussed in climatic and intrinsic contexts.

Turtles nest in Vamizi all year long. However, the two main beaches of Farol and Comissete show different nesting peaks, although at the same beach, they differ in duration between years, showing some irregularity. At the Vamizi beaches of Farol and Comissete, green turtles show a similar pattern of nesting seasonality found in the adjacent islands of Eparses. On Grande Glorieuse, the nesting season is longer and shows a more variable peak, which happens in the dry season months (March to June; Lauret-Stepler et al. 2007), a similar pattern found for Farol (February-June) during the period 2003 – 2007. This pattern was observed at Juani Island (Tanzania), on its eastern side, where nesting activity happens all year long with a more restrictive peak in April and May (West et al. 2013). However, at Farol beach the nesting peak is different in the later study period (2008 – 2010), with the highest nesting activity observed between March and July. The delaying of the nesting period at Farol may be due to behavioral flexibility (Hawkes et al. 2009). As noted by several authors, turtles seem to choose nest locations (Cheng et al. 2013; Hamann et al. 2007) based on sensitivities to changes in their environment and show a biological capacity to adapt to climate crises (Fuentes et al. 2010). The nesting pattern observed in Comissete beach is similar to that published by Lauret-Stepler et al. (2007) in Tromelin and Europa islands (inside MZC), with a more stable peak at the wet season (November to February).

#### Inter-nesting period and remigration interval

From published literature, the modal inter-nesting value for green is 12 days (range: 10 – 17 days; Almeida et al. 2011), which is similar with what we observed in Vamizi. In Mayotte Island, Bourjea et al. (2007a) argue for the existence of three inter-nesting peaks: the first between 1 and 7 days (corresponding to 25% of females that abort their first nesting attempt); the second between 12 and 14 days; and the third between 25 and 28 days (third nest attempt for the same female); though bigger intervals can happen over 150 days. This analysis is very similar to what we observed in Vamizi, though we also find that the second emergence can happen in a longer period.

The standard deviation of the mean inter-nesting period of Vamizi is also bigger compared with values obtained by Bourjea et al. (2007a) and West et al. (2013). The re-migration interval (2.6 ± 1.1 years) obtained for Vamizi is consistent with data from Bourjea et al. (2007a). This result emphasizes that the WIO population maintains an expected behavior regarding migration cycles between foraging and nesting grounds.

#### Nesting females and clutch frequency

The estimated number of nesting females/year between 2008 and 2010 was low (52), which makes Vamizi a small rookery. Garnier et al. (2012) predicted that Vamizi received approximately 50 females per year during 2004 – 2007, which was similar to estimations for the following years; though 2009 had a smaller number of visitors. Associated with the reduction in specimen size, this number can be an indication of regular recruitment needed to maintain the number of reported nesting females. The observed clutch frequency averages (2008 – 2010) were higher than those reported by Garnier et al. (2012) for the previous period (2004 – 2007).

#### Clutch size, hatching and emergence successes

The observed average clutch size (116.7 ± 26.5 eggs) falls between standard published values that range between 110 and 130 eggs (Pritchard and Mortimer 1999). Observed values also fall in the range found for other beaches/locations on the WIO: 78 – 120 eggs.nest^-1^ (Waqas et al. 2011), 116 ± 24 eggs (Innocenzi et al. 2010), 134 ± 14 eggs (West et al. 2013).

Hatching and emergence successes in Vamizi are higher than in Mohéli (Innocenzi et al. 2010) and in Juani Island, at the Tanzanian coast (West et al. 2013). Environmental factors, such as temperature (Davenport 1997), rainfall (Matsuzawa et al. 2002), erosion (Mazaris et al. 2009), and sea level rise (Fuentes et al. 2011; Hawkes et al. 2009) may influence several nesting parameters like hatching and emergence successes. Yet, erosion and changes in sea level rise are linked with climate change and are enhanced by extreme weather events (Van Houtan and Bass 2007; Hawkes et al. 2009). Other intrinsic factors, such as sand type (Hays et al. 2001; Fuentes et al. 2010), predators (Brown and Macdonald 1995; Mendonça et al. 2010), and human pressures (Mazaris et al. 2009; Antworth et al. 2006) interfere with these nesting parameters.

The rainfall variability of 2004 – 2010 and its influence on sand heat conductivity may play a role in the incubation period differences, especially at Farol beach. Sand characteristics are known to influence the sand temperature, and consequently the duration of egg incubation (Hawkes et al. 2009). Vamizi beaches are composed of biogenic sand, which is formed by *in situ* accumulation of short-distance transportation hermatypic coral reef and other marine organism debris (-0.15 – 0.35 – mm). Compared with to quartz sands, coral sands show different mechanical properties: high grain density, high porosity (ranging from 0.54 to 2.97, which is much higher than quartz sand porosity), high fragmentation and low psephisity (Chengjie et al. 2013). Lin et al. (2008) show that the water content of soil is one important factor that affects its thermal resistance. Therefore in Vamizi the precipitation may be influencing changes in incubation conditions and affecting its duration, which may explain the statistical differences found between rainy and dry years (like 2005). However, these sands are able to maintain incubation properties that result in high hatching and emergence rates. These variations may be normal, since they are linked to the seasonality created by the monsoons.

#### Incubation period

In the last years of the study, Farol showed a decreasing tendency of its incubation period and the peak of the number of nests moved from April (2003 – 2007) to May (2008 – 2010). Incubation period is dependent on temperature (see Hawkes et al. 2009 and Davenport 1997), and the sex of hatchling is determined by the incubation temperature (Davenport 1997). The pivotal temperature of 29.2°C (Broderick et al. 2000; Godfrey and Mrosovsky 2006) will determine the sex, with males being produced below that temperature and females being produced above. A pivotal incubation period has also been estimated to be 56 days (Broderick et al. 2000). The observed values for the incubation period are above this pivotal period, indicating that the sex ratio could be male-biased. Models by Fuentes et al. (2010) predict that “climate change will increase sand temperature at the nesting grounds”, which will result in the feminization of hatchlings by 2030. However, the MZC is very different regarding climatic responses. In their study of the link between the precipitation field and ocean dynamics, Saji et al. (1999) report that the air-sea interaction process is different in the WIO, for example, it is independent of the El Niño/Southern Oscillation (ENSO). This means that other climatic-sea water features are possibly affecting the behavioral patterns seen in this study from Vamizi green turtles. Most likely the stability of the NE/SE monsoons is an important factor in green turtle nesting behavior in the northern area of the MZC.

#### Flooded nests – Sea -level rise and its influence in nesting season at Vamizi

Rising sea-levels may also be influencing Vamizi beaches rookeries. Garnier et al. (2012) suggest that nest loss through inundation that occurred in Vamizi in 2007, emphasizes that the phenomena might “be indicative of a regional or global event”. The Maziwi Island in Tanga (Tanzania), known for having been “the most important single turtle nesting ground in east Africa”, was entirely submerged in 1978 (Mahongo 2009). While clearing the vegetation on the island accelerated the process, other factors such as, erosion from storms, or sea level rise may have also played a part (Mahongo 2009). Through local observations it is clear that Vamizi is suffering from erosion, which means there is an urgent need to monitor sea level trends at the site. This kind of information is lacking not only in Mozambique, but also in the WIO (Mahongo 2009). To mitigate future problems related to global rise in sea-level, it is important to understand the phenomena locally, for example, what will be the extent of nesting grounds being affected in the next 10 years in the rookeries of the north MZC. The observed tendency over 60 years of data collection and data analysis from Indian Ocean showed an average sea level trend rise of 3.4 ± 0.7 mm per year (1953 – 2009; Palanisamy et al. 2014). The beaches of Farol and Comissete may provide an important study ground to monitor water/air parameters, and the effects that changes in these may have on female’s behavior/choices because they are small but efficient nesting grounds at the present. They seem to be also an important point of dispersal of turtles to the north of the coast, and to the south, entering deeper in the MZC. From 2009 to 2011, Bourjea et al. (2013) tracked nesting green turtle females to the nesting peak in the rookeries of Europa, Glorieuses, Tromelin, Mayotte, and Mohéli, using 81 satellite transmitters. Their results revealed the migratory pathways of these females in the WIO, showing that 39.7% of them used Madagascar as a costal foraging ground, and others used Mozambique, Kenya, Tanzania and Somalia coasts. They emphasize that the extreme north of Madagascar functions as an important coastal migratory corridor (Bourjea et al. 2013). Garnier et al. (2012), using satellite transmitters, showed the migration routes of four green turtle females tagged in Vamizi, in the direction of foraging grounds situated in Tanzania, Kenya and northwest Madagascar (Nosy Makamby).

#### Genetic diversity

Haplotype diversity at Vamizi is considered high and similar to that already described for this region (Formia et al. 2006; Bourjea et al. 2007b). With the use of a 1000 bp fragment of the mitochondrial DNA control region it was possible to identify 11 haplotypes, seven of them new.

Haplotypes IND1 and IND3 are present in Vamizi and were also reported on the Comoros by Formia et al. (2006). The Comoros are very near Vamizi, and they play an important role in the currents at the north of the MZC (Ternon et al. 2014), where an intermittent gyre is generated around them (Obura et al. 2012). It forces water, at intermediate depths, to circulate eastward rather than entering directly into the northern part of the Channel (Ternon et al. 2014), perhaps enabling, female turtles to visit nesting beaches around.

The haplotype CMJ1 occurs in the Western Pacific, in a foraging site for green turtles (Hamabata et al. 2009). Though its frequency was very low in our study, CMJ1 nevertheless shows a connection between the Eastern Indian (EIO) and Western Pacific Oceans. Formia et al. (2006) aligned IND haplotypes with haplotypes from the western Pacific and concluded that they show high similarity, corroborating the link between WIO and the western Pacific.

The CM8 haplotype, detected in high frequency at Vamizi, occurs mainly in the Atlantic (Formia et al. 2006). The CM8 haplotype was also identified in southwest Indian Ocean rookeries (Europe, Juan de Nova) and at lower frequencies in Mayotte and Mohéli (Bourjea et al. 2007b). Bourjea et al. (2007b) found that the CM8 frequency decreases from the south of the MZC towards the north of the MZC rookeries (Bourjea et al. 2007b). These authors also suggest that the existence of the CM8 haplotype may be indicative of an active dispersal of green turtles from the Atlantic into the Indian Ocean waters, by the Cape of Good Hope an possibility also raised by Shamblin et al. (2014) for loggerhead turtles.

Luschi et al. (2006) tracked the journey of post nesting leatherbacks by satellite between 1996 and 2003, from their nesting site at the Maputaland coast of KwaZulu-Natal of southeastern Africa, and showed that two females entered the southeast Atlantic Ocean, which demonstrated that turtles can migrate between MZC and Atlantic Ocean waters.

Despite its high turbulence waters, the MZC doesn’t seem to function has a natural barrier because artificial drifters were moved (by currents) northwards, inside the Channel, against the southward migration eddy field (see Hancke et al. 2014). This probably explains the presence of CM8 in Vamizi and adjacent islands, being brought by females that migrate northwards. However information is still lacking on Indian Ocean phylogenies and so further research is needed. Obura et al. (2012) states that, “genetic differences could result from oceanographic features that affect the movements of juveniles”, but how? Part of the juveniles may be dragged by mesoscale eddies towards the south of MZC, and others may be dragged by the EACC, to the north of the WIO (to the foraging areas at Tanzania, Kenya, etc.). The behavior of philopatry may bring some of those juveniles later, as nesting females, to rookeries like Vamizi, which would explain its high haplotype diversity and the existence of haplotypes from the south and from the north of the WIO. It is important to understand the differences between the Vamizi population and populations found by other investigators in the area. For example, does it represent a different genetic sub-population from the females nesting in Europe Island, Mayotte, Comoros, Nosy Iranja, Glorieuses and Aldabra described by Bourjea et al. (2007b)? The observed migratory pathways, both coastal and pelagic, that encompasses the East African coast through Tanzania and Kenya and the Northwest of Madagascar (Garnier et al. 2012; Bourjea et al. 2013) seem to corroborate this possibility.

#### Conservation

Our study in Vamizi showed that, despite receiving nesting females in two periods of the year during 2003 – 2010, the distribution of nesting activity has decreased since 2005 at Comissete beach with a proportional increase in Farol beach. This may reveal an adaptation of female behavior towards anthropogenic factors. In Comissete, associated to the opening of a lodge in 2005, beach sand was mixed and cleaned and human presence and activity increased. Turtles are sensitive to human presence on their nesting beaches (Antworth et al. 2006) and are known to move to nearby areas, lacking human presence (Weishampel et al. 2003). Based on the observed nests, the implementation of the touristic project, including the construction of infrastructures in Farol can change the importance of each nesting beach. Though paleontological records show their resilience and capacity for adaptation to geologic/climatic changes (Fuentes et al. 2010), it has not been predicted the effect of human pressures combined with climatic fluctuations on their survival. However, it has been discovered that green turtles are capable of breaking their natal philopatry and choosing alternative nesting grounds (Fuentes et al. 2010).

It has been shown that a nesting beach can be abandoned within 40 years (one turtle generation; Fuentes et al. 2010), thus changing the spatial distribution of nesting and foraging grounds. It would be important to decipher which pressures are more likely to induce changes in green turtle behaviors in the northern MZC, which will lead to changes in migration routes, and breeding sites.

Vamizi Island is included in the *Mtwara-Quirimbas Complex*, a priority site for conservation identified in WWF Eastern African Marine Ecoregion (Rosendo et al. 2011). As defended by the report “Assessing Marine World Heritage from an Ecosystem Perspective: The Western Indian Ocean” the proposed site with Outstanding Universal Value (OUV) of “Northern Mozambique to southern Tanzania – Nacala – Quirimbas – Mtwara” must be sufficiently assessed to meet the strict criteria to be designated as World Heritage (Obura et al. 2012). Also, the World Bank has been supporting projects like the “Coastal and Marine Biodiversity Management Project”, which aim to protec areas in northern Mozambique, by supporting studies to establish a marine protected area (MPA), the Rovuma National Reserve, that awaits government approval (Rosendo et al. 2011). Should it be approved, Vamizi will be included in a privileged location where the marine turtle program initiated in 2002. The continuity of the monitoring and conservation program will help to accomplish the goal to qualify it as an OUV area.

## Conclusions

Vamizi beaches host approximately 52 nesting females per year, which have been showing a reduction in their length over time. This may be a sign that cohorts of younger turtles are being removed from the population, during the inter-nesting period and/or between the migrations from the foraging to the nesting grounds. This observation is coherent with the need raised by several authors for international cooperation for the protection of marine turtles in the WIO.

This study contributes information on the genetic diversity of a sample of nesting turtles in Vamizi; information that was previously lacking in the literature. The characterization of the diversity based on longer (850 bp) control region sequences enabled a better separation of the haplotypes that may not have been identified in previous analysis. This information may help to provide a redefinition of regional units of conservation. The genetic diversity and high rates of hatching and emergence success demonstrate that Vamizi Island is an important site for producing and dispersing diverse hatchlings.

This Island is situated in an area with high marine biodiversity and with proven success in incubating turtle eggs. It is possible that the characteristics of the biogenic sand can explain the high rates of hatching and emergence success obtained and is consequently critical for conservation. The sand characteristics may help to minimize ambient changes (e.g. temperature), which are known to affect nesting and incubation parameters.

The main concerns regarding the Vamizi Island rookery are reduction of incubation period values, dislocation of peak nesting activity, increase in the number of flooded nests in the later years of this study and human pressure. Future research is needed to understand the factors that are leading to these changes.

The possibility that turtles react to human activity must be considered in infrastructure planning, especially for touristic purposes, near beaches with importance as nesting grounds, like Comissete and Farol. Some resilience and behavioral plasticity in sea turtles seems to occur regarding human territory occupancy and climate changes.

### Ethics

The data was collected according published methodologies (see Eckert et al. 1999) and by qualified personnel and in authorized areas.

## Electronic supplementary material

Additional file 1: Table S1: Nucleotide sequences and the 74 polymorphic sites identified on the green turtle. (DOCX 83 KB)

## References

[CR1] Almeida AP, Moreira LMP, Bruno SC, Thomé JCA, Martins AS, Bolten AB, Borndal KA (2011). Green turtle nesting on Trindade Island, Brazil: abundance, trends, and biometrics. Endang Species Res.

[CR2] Alvarado J, Murphy TM, Eckert KL, Bjorndal KA, Abreu-Grobois FA, Donnelly M (1999). Nesting Periodicity and Internesting Behavior. Research and Management Techniques for the Conservation of Sea Turtles.

[CR3] Antworth RL, Pike DA, Stiner JC (2006). Nesting ecology, current status, and conservation of sea turtles on an uninhabited beach in Florida. USA Biol Conserv.

[CR4] Avise JC, Bowen BW, Lamb T, Meylan AB, Bermingham E (1992). Mitochondrial DNA evolution at a turtle’s pace: evidence for low genetic variability and reduced microevolutionary rate in the testudines. Mol Biol Evol.

[CR5] Bagda E, Bardakci F, Turkozan O (2012). Lower genetic structuring in mitochondrial DNA than nuclear DNA among the nesting colonies of green turtle (*Chelonia mydas*) in the Mediterranean. Biochemical Systematics and Ecology.

[CR6] Balazs GH, Eckert KL, Bjorndal KA, Abreu-Grobois FA, Donnelly M (1999). Factors to Consider in the Tagging of Sea Turtles. Research and Management Techniques for the Conservation of Sea Turtles.

[CR7] Bjorndal KA, Bolten AB, Tröeng S (2005). Population structure and genetic diversity in green turtles nesting at Tortuguero, Costa Rica, based on mitochondrial DNA control region sequences. Mar Biol.

[CR8] Blanco GS, Morreale SJ, Bailey H, Seminoff JA, Paladino FV, Spotila JR (2012). Post-nesting movements and feeding grounds of a resident East Pacific green turtles *Chelonia mydas* population from Costa Rica. Endang Species Res.

[CR9] Bolten AB, Eckert KL, Bjorndal KA, Abreu-Grobois FA, Donnelly M (1999). Techniques for Measuring Sea Turtles. Research and Management Techniques for the Conservation of Sea Turtles.

[CR10] Bourjea J, Frappier J, Quillard M, Ciccione S, Roos D, Hughes G, Grizel H (2007). Mayotte Island: another important green turtle nesting site in the southwest Indian Ocean. Endang Species Res.

[CR11] Bourjea J, Lapègue S, Gagnevin L, Broderick D, Mortimer JA, Ciccione S, Roos D, Taquet C, Grizel H (2007). Phylogeography of the green turtle, *Chelonia mydas*, in the Southwest Indian Ocean. Mol Ecol.

[CR12] Bourjea J, Nel R, Jiddawi NS, Koonjul MS, Bianchi G (2008). Sea Turtle Bycatch in the West Indian Oceans: review, recommendations and research priorities. Western Indian Ocean J Mar Sci.

[CR13] Bourjea J, Ciccione S, Behamou S, Dalleau M (2013). Etude in situ de la migration post-reproductive des femelles de tortues vertes (Chelonia mydas) dans l’océan Indien Occidental. Indian Ocean Tuna Commission, IOTC-WPEB09-25.

[CR14] Broderick AC, Godley BJ, Reece S, Downie JR (2000). Incubation periods and sex ratios of green turtles: highly female biased hatchling production in eastern Mediterranean. Mar Ecol Prog Ser.

[CR15] Brown L, Macdonald W (1995). Predation on green turtle *Chelonia mydas* nests by wild canids at Akyatan beach, Turkey. Biological Conservation.

[CR16] Cheng IJ, Bentivegna F, Hochscheid S (2013). The behavioural choices of green turtles nesting at two environmentally different islands in Taiwan. J Exp Mar Biol Ecol.

[CR17] Chengjie Z, Peidong LU, Yanhong W (2013). Proceedings of the 7th International Conference on Asian and Pacific Coasts (APAC 2013). Experiment Study On Physical Properties And Motional Characteristics of Coral Sand.

[CR18] Costa A, Motta H, Pereira MAM, Videira EJS, Louro CMM, João J (2007). Marine Turtles in Mozambique: towards an effective conservation and management program. Mar Turt Newsl N°.

[CR19] Davenport J (1997). Temperature and the life-history strategies of sea turtles. J Therm Biol.

[CR20] Dutton PH, Bowen BW, Witzell WN (1996). Methods for Collection and Preservation of Samples for Sea Turtle Genetic Studies. Proceedings of the International Symposium on Sea Turtle Conservation Genetics.

[CR21] Dutton PH, Balazs GH, LeRoux RA, Murakawa SKK, Zarate P, Sarti Martínez L (2008). Composition of Hawaiian green turtle foraging aggregations: mtDNA evidence for a distinct regional population. Endang Species Res.

[CR22] Eckert KL, Bjorndal KA, Abreu-Grobois FA, Donnelly M (1999). Research and Management Techniques for the Conservation of Sea Turtles.

[CR23] Encalada SE, Lahanas PN, Bjorndal KA, Bolten AB, Miyamoto MM, Bowen BW (1996). Phylogeography and population structure of the Atlantic and Mediterranean green turtle (*Chelonia mydas*): a mitochondrial DNA control region sequence assessment. Mol Ecol.

[CR24] Formia A, Godley BJ, Dontaine J-F, Bruford MW (2006). Mitochondrial DNA diversity and phylogeography of endangered green turtle (*Chelonia mydas*) populations in Africa. Conserv Genet.

[CR25] Formia A, Broderick AC, Glen F, Godley BJ, Hays GC, Bruford MW (2007). Genetic composition of the Ascension Island green turtle rookery based on mitochondrial DNA: implications for sampling and diversity. Endangered Species Research.

[CR26] Fuentes MMPB, Hamann M, Limpus CJ (2010). Past, current and future thermal profiles of green turtle nesting grounds: implications from climate change. J Exp Mar Biol Ecol.

[CR27] Fuentes MMPB, Limpus CJ, Hamann M (2011). Vulnerability of sea turtle nesting grounds to climate change. Glob Chang Biol.

[CR28] Garnier J, Hill N, Guissamulo A, Silva I, Witt M, Godley B (2012). Status and community-based conservation of marine turtles in the northern Querimbas islands (Mozambique). Oryx.

[CR29] Godfrey MH, Mrosovsky N (2006). Pivotal Temperature for green sea turtles, *Chelonia mydas*, nesting in Suriname. Herpetol J.

[CR30] Godley BJ, Broderick AC, Hays GH (2001). Nesting of green turtles (*Chelonia mydas*) at Ascension Island, South Atlantic. Biol Conserv.

[CR31] Godley BJ, Barbosa C, Bruford M, Broderick AC, Catry P, Coyne MS, Formia A, Hays GC, Witt MJ (2010). Unravelling migratory connectivity in marine turtles using multiple methods. J Appl Ecol.

[CR32] Hamabata T, Nishida S, Kamezaki N, Koike H (2009). Genetic structure of populations of the green turtle (*Chelonia mydas*) in Japan using mtDNA control region sequences. Bulletin of the Graduated School of Social and Cultural Studies, Kyushu University.

[CR33] Hamann M, Limpus CJ, Read MA, Johnson JE, Marshall P (2007). Part II: Species and Species Groups, Chapter 15 Vulnerability of Marine Reptiles in the Great Barrier Reef to Climate Change. Climate Change and the Great Barrier Reef.

[CR34] Hancke L, Roberts MJ, Ternon JF (2014). Surface drifter trajectories highlight flow pathways in theMozambiqueChannel. Deep-Sea Research II.

[CR35] Hawkes LA, Broderick AC, Godfrey MH, Godley BJ (2009). Climate change and marine turtles. Endang Species Res.

[CR36] Hays GC, Ashworth JS, Barnsley MJ, Broderick AC, Emery DR, Godley BJ, Henwood A, Jone EL (2001). The importance of sand albedo for the thermal conditions on sea turtle nesting beaches. Oikos.

[CR37] Hughes G (1971). Preliminary report on the sea turtles and dugongs of Mozambique. Veterinária Moçambicana, Lourenço Marques.

[CR38] Innocenzi J, Maury J, M’soili A, Ciccione S (2010). Reproduction biology of green turtle in Itsamia, Mohéli (Union of Comoros). Indian Ocean Turtle Newslett N°.

[CR39] Lahanas PN, Bjorndal KA, Bolten AB, Encalada SE, Miyamoto MM, Valverde RA, Bowen BW (1998). Genetic composition of green turtle (*Chelonia mydas*) feeding ground population: evidence for multiple origins. Mar Biol.

[CR40] Lara-Ruiz P, Lopez GG, Santos FR, Soares LS (2006). Extensive hybridization in hawksbill turtles (*Eretmochelys imbricata)* nesting in Brazil revealed by mtDNA analyses. Conserv Genet.

[CR41] Lauret-Stepler M, Bourjea J, Roos D, Pelletier D, Ryan PG, Ciccione S, Grizel H (2007). Reproductive seasonality and trend of *Chelonia mydas* in the SW Indian Ocean: a 20 yr study based on track counts. Endang Species Res.

[CR42] Lauret-Stepler M, Ciccione S, Bourjea J (2010). Monitoring of marine turtles reproductive activities in Juan de Nova, Eparses Islands, South Western Indian Ocean, based on tracks count and width. Indian Ocean Turtle Newslett N°.

[CR43] Lee PLM (2008). Molecular ecology of marine turtles: new approaches and future directions. J Exp Mar Biol Ecol.

[CR44] Lee PM, Luschi P, Hays GC (2007). Detecting female precise natal philopatry in green turtles using assignment methods. Mol Ecol.

[CR45] Lewison RL, Crowder LB, Read AJ, Freeman SA (2004). Understanding impacts of fisheries bycatch on marine megafauna. Trends Ecol Evol.

[CR46] Limpus CJ, Leisa F (2008). A Biological Review of Australian Marine Turtle Species. 2. Green turtle, *Chelonia mydas* (Linnaeus). Biological Review of Australian Marine Turtles Species, Environmental Protection Agency.

[CR47] Lin C-K, Kulasiri D, Chien L-K (2008). Soil Thermal Conductivity Study in Western Coastal Zone of Taiwan.

[CR48] Luschi P, Lutjeharms JRE, Lambardi P, Mencacci R, Hughes GR, Hays GC (2006). A review of migratory behaviour of sea turtles off southeastern Africa. S Afr J Sci.

[CR49] Mahongo SB (2009). The changing global climate and its implications on sea level trends in Tanzania and the Western Indian Ocean Region. Western Indian Ocean Journal Marine Science.

[CR50] Matsuzawa Y, Sato K, Sakamoto W, Bjorndal KA (2002). Seasonal fluctuations in sand temperature: effects on the incubation period and mortality of loggerhead sea turtle (*Caretta caretta*) pre-emergent hatchlings in Minabe, Japan. Mar Biol.

[CR51] Mazaris AD, Matsinos G, Pantis JD (2009). Evaluating the impacts of coastal squeeze on sea turtle nesting. Ocean Coast Manage.

[CR52] McClanahan T (1988). Seasonality in East Africa’s coastal waters. Mar Ecol-Prog Ser.

[CR53] Mendonça VM, Saady SA, Kiyumi AA, Erzini K (2010). Interactions between Green Turtles (*Chelonia mydas*) and Foxes (*Vulpes vulpes arabica*, *V. rueppellii sabaea*, and *V. cana*) on Turtle Nesting Grounds in the Northwestern Indian Ocean: Impacts of the Fox Community on the Behavior of Nesting Sea Turtles at the Ras Al Hadd Turtle Reserve, Oman. Zool Stud.

[CR54] Meylan AB, Bowen BW, Avise JC (1990). A genetic test of the natal homing versus social facilitation models for green turtle migration. Science.

[CR55] Miller JD, Eckert KL, Bjorndal KA, Abreu-Grobois FA, Donnelly M (1999). Determining Clutch Size and Hatching Success. Research and Management Techniques for the Conservation of Sea Turtles.

[CR56] Missão Hidrográfica de Moçambique (MHM) (1974). Canal de Moçambique, Estado de Moçambique, Carta hidrográfica de Palma à Ilha Vamizi, 1955–1972. 1ª Ed., n° 451, Escala 1/50 000.

[CR57] Monzón-Argüello C, Rico C, Marco A, López P, López-Jurado LF (2010). Genetic characterization of eastern Atlantic hawksbill turtles at a foraging group indicates major undiscovered nesting populations in the region. J Exp Mar Biol Ecol.

[CR58] Mortimer JA (2002). A strategy to conserve and manage the sea turtle resources of the Western Indian Ocean region.

[CR59] Narane DA, Videira EJS, Pereira MAM, Louro CMM, Narane DA (2008). Cabo de São Sebastião. Monitoria, Marcação e Conservação de Tartarugas Marinhas em Moçambique: Dados Históricos e Relatório anual 2007/08.

[CR60] Narane DA, Videira EJS, Pereira MAM, Louro CMM, Narane DA (2008). Parque Nacional do Arquipélago do Bazaruto. Monitoria, Marcação e Conservação de Tartarugas Marinhas em Moçambique: Dados Históricos e Relatório anual 2007/08.

[CR61] Obura DO, Church JE, Gabrié C (2012). Assessing Marine World Heritage from an Ecosystem Perspective: The Western Indian Ocean.

[CR62] Palanisamy H, Cazenave A, Meyssignac B, Soudarin L, Wöppelmann G, Becker M (2014). Regional sea level variability, total relative sea level rise and its impacts on islands and coastal zones of Indian Ocean over the last sixty years. Glob Planet Chang.

[CR63] Piniak WED, Eckert KL (2011). Sea turtle nesting habitat in the Wider Caribbean Region. Endanger Species Res.

[CR64] Pritchard PCH, Mortimer JA, Eckert KL, Bjorndal KA, Abreu-Grobois FA, Donnelly M (1999). Taxonomy, External Morphology and Species Identification. Research and Management Techniques for the Conservation of Sea Turtles.

[CR65] Richmond ME (2002). A Field Guide to the Seashores of Eastern Africa and the Western Indian Ocean Islands.

[CR66] Rosendo S, Brown K, Joubert A, Jiddawi N, Mechisso M (2011). A clash of values and approaches: a case study of marine protected area planning in Mozambique. Ocean Coast Manage.

[CR67] Saji NH, Goswami BN, Vinayachandran PN, Yamagata T (1999). A dipole mode in the tropical Indian Ocean. Nature.

[CR68] Sambrook J, Fritsch EF, Maniatis T (1989). Molecular Cloning: A Laboratory Manual, 2nd edn.

[CR69] Schroeder B, Murphy S, Eckert KL, Bjorndal KA, Abreu-Grobois FA, Donnelly M (1999). Population Surveys (ground and aerial) on Nesting Beaches. Research and Management Techniques for the Conservation of Sea Turtles.

[CR70] Shamblin BM, Bolten AB, Abreu-Grobois FA, Bjorndal KA, Cardona L, Carreras C, Clusa M, Monzón-Argüello C, Nairn CJ, Nielsen JT, Nel R, Soares LS, Stewart KR, Vilaça ST, Türkozan O, Yilmaz C, Dutton PH (2014). Geographic patterns of genetic variation in a broadly distributed marine vertebrate: new insights into loggerhead turtle stock structure from expanded mitochondrial DNA sequences. PLoS ONE.

[CR71] Shanker K (2004). Marine turtles status and conservation in the Indian Ocean. Paper presented at the Expert Consultation on the interactions between Sea Turtles and Fisheries within an Ecosystem Context.

[CR72] Ternon JF, Roberts MJ, Morris T, Hancke L, Backeberg B (2014). *In situ* measured current structures of the eddy field in the Mozambique Channel. Deep-Sea Research II.

[CR73] Thompson JD, Higgins DG, Gipson TJ (1994). CLUSTAL W: improving the sensitivity of progressive multiple sequence alignment through sequence weighting, position-specific gap penalties and weight matrix choice. Nucleic Acids Res.

[CR74] Tröeng S, Evans D, Harrison E, Lagueux CJ (2005). Migration of green turtles *Chelonia mydas* from Tortuguero, Costa Rica. Mar Biol.

[CR75] Van Houtan KS, Bass OL (2007). Stormy oceans are associated with declines in sea turtle hatching. Curr Biol.

[CR76] Videira EJS, Pereira MAM, Louro CMM, Narane DA (2008). Monitoria, Marcação e Conservação de Tartarugas Marinhas em Moçambique: Dados Históricos e Relatório anual 2007/08.

[CR77] Wallace BP, Lewison RL, McDonald SL, McDonald RK, Kot CY, Kelez S, Bjorkland RK, Finkbeiner EM, Helmbrecht S, Crowder LB (2010). Global patterns of marine turtle bycatch. Conserv Lett.

[CR78] Walsh PS, Metzer DA, Higuchi R (1991). Chelex–100 as a medium for simple extraction of DNA for PCR-based typing from forensic material. Biotechniques.

[CR79] Waqas U, Hasnain SA, Ahmad E, Abbasi M, Pandrani A (2011). Conservation of Green Turtle (*Chelonia mydas)* at Daran Beach, Jiwani, Balochistan. Pakistan J Zool.

[CR80] Weishampel JF, Bagley DA, Ehrhart LM, Rodenbeck BL (2003). Spatiotemporal patterns of annual sea turtle nesting behaviours along an East Central Florida beach. Biol Conserv.

[CR81] West L, Mochomvu B, Abdullah O, Mapoy S (2013). Green turtle nesting activity at Juani Island, Tanzania, during the 2012 peak nesting season. Indian Ocean Turtles Newslett.

