# Supplementary material for: Reproductive biology and genetic diversity of the green turtle (Chelonia mydas) in Vamizi island, Mozambique
Source: Springerplus. 2014 Sep 19;3:540. doi: 10.1186/2193-1801-3-540 (PMC4447848; doi:10.1186/2193-1801-3-540)
Supplement: Supplementary file 1 — Additional file 1: Table S1: Nucleotide sequences and the 74 polymorphic sites identified on the green turtle. (DOCX 83 KB) [file 40064_2014_1558_MOESM1_ESM.docx]

Table S1. Nucleotide sequences and the 74 polymorphic sites identified on the green turtle.
